# Advanced models of human skeletal muscle differentiation, development and disease: Three-dimensional cultures, organoids and beyond

**DOI:** 10.1016/j.ceb.2021.06.004

**Published:** 2021-12

**Authors:** Salma Jalal, Sumitava Dastidar, Francesco Saverio Tedesco

**Affiliations:** 1 Department of Cell and Developmental Biology, University College London, WC1E 6DE London, United Kingdom; 2 The Francis Crick Institute, 1 Midland Road, London NW1 1AT, United Kingdom; 3 Dubowitz Neuromuscular Centre, Great Ormond Street Institute of Child Health, University College London, London WC1N 1EH, United Kingdom; 4 Department of Paediatric Neurology, Great Ormond Street Hospital for Children, WC1N 3JH London, United Kingdom

**Keywords:** Skeletal muscle, Stem cells, iPS cells, 3D cultures, Organoids, Tissue engineering, Disease modelling

## Abstract

Advanced *in vitro* models of human skeletal muscle tissue are increasingly needed to model complex developmental dynamics and disease mechanisms not recapitulated in animal models or in conventional monolayer cell cultures. There has been impressive progress towards creating such models by using tissue engineering approaches to recapitulate a range of physical and biochemical components of native human skeletal muscle tissue. In this review, we discuss recent studies focussed on developing complex *in vitro* models of human skeletal muscle beyond monolayer cell cultures, involving skeletal myogenic differentiation from human primary myoblasts or pluripotent stem cells, often in the presence of structural scaffolding support. We conclude with our outlook on the future of advanced skeletal muscle three-dimensional cultures (e.g. organoids and biofabrication) to produce physiologically and clinically relevant platforms for disease modelling and therapy development in musculoskeletal and neuromuscular disorders.

## Introduction

**Figure 1:**
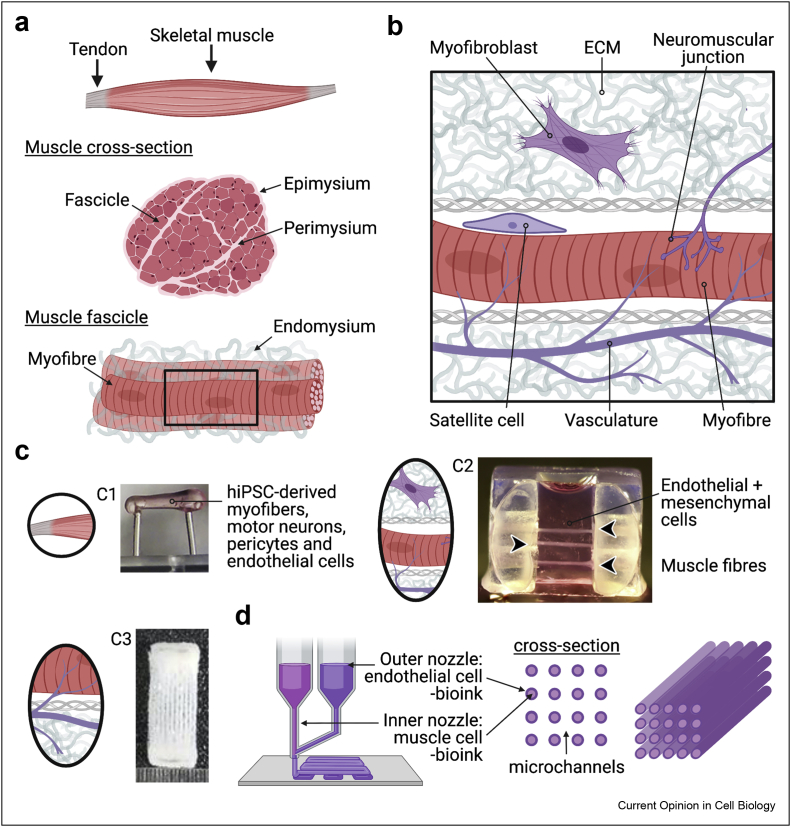
**Recreating human skeletal muscle architecture *in vitro*.** Created with BioRender.com. **(a)** Physiological structure of human skeletal muscle tissue. **(b)** Schematic of the skeletal muscle tissue niche including myofibres, vasculature, satellite cells, myofibroblasts, motor neuron endings (in the neuromuscular junction) and extracellular matrix (ECM). **(c)** Selected examples of culture platforms that integrate different components of the skeletal muscle tissue niche. C1) humaninduced pluripotent stem cell (hiPSC) derived cells (including myofibres, motor neurons, pericytes and endothelial cells) are differentiated in a fibrin hydrogel held under tension between two silicone posts; copyright 2018 Elsevier [40]. C2) myoblasts were first formed into myotubes in a fibrin gel that were subsequently surrounded by a fibrin gel solution containing endothelial cells and fibroblasts; copyright 2018 Elsevier [55]. C3) 3D bioprinted cylindrical tubes of myogenic progenitors with or without encasement by an endothelial cell layer in parallel with hollow microchannels**;** copyright 2019 Elsevier [59]. **(d)** Illustration of a coaxial 3D bioprinting setup. 3D, three-dimensional.

The skeletal muscle, an architecturally complex tissue that accounts for the largest tissue mass in the human body, is responsible for supporting posture, voluntary movement, guarding soft tissues and body openings, as well as regulating several metabolic and homoeostatic functions. Functional skeletal muscle not only contains myofibres and their progenitor cells but also requires their constant interaction with other cell types and tissues including, but not limited to, connective tissue, vasculature and motor neurons [1]. The hierarchical organisation of skeletal muscle (Figure 1a) consists of organised bundles of fascicles which in turn are composed of bundles of myofibres embedded within three layers of extracellular matrix (the endomysium, perimysium and epimysium) [2]. The importance of the interplay between different compartments of the skeletal muscle niche (Figure 1b) is exemplified on acute injury, when multiple mechanisms are initiated within the different compartments that eventually converge to activate tissue-resident muscle stem cells (MuSCs, also known as satellite cells). For instance, damaged blood vessels can release cytokines [3] or inflammatory cells [4] to support regeneration at an injury site.

Normal tissue function and repair/regeneration can be overcome in large acute muscle injuries as well as in chronic severe musculoskeletal disorders such as muscular dystrophy [5], where different components of the skeletal muscle tissue functional units and niche are compromised. Given ethical considerations and limited tissue availability, it is often difficult to study skeletal muscle developmental dynamics, regeneration and disease pathogenesis in human subjects or their biopsies. Although traditional cell culture and animal models have been used to elucidate some molecular aspects behind these processes, limitations in using different species [6] and systems lacking physiologically relevant extracellular cues [7] make it difficult to translate such findings to the human context. Bioengineering human models with higher fidelity to native skeletal muscle tissues can overcome these limitations and enable researchers to advance our fundamental understanding of the mechanistic processes behind muscle development and regeneration. Such insights can be further applied to disease modelling, biomarker detection, drug screening and regenerative medicine.

In this review, we will start with a brief overview of skeletal myogenic cell generation and differentiation followed by a discussion on recently developed three-dimensional (3D) platforms, developed with human biopsy-derived myoblasts (primary or immortalised) or pluripotent stem cells. We then conclude with our perspectives on the future of artificial skeletal muscle models by discussing methods to develop physiologically complex models able to deliver clinically relevant phenotypic readouts that can be used as outcome measures for therapy development. We will not highlight studies based on platforms using rodent myogenic cells, nor those involving top-down approaches such as tissue decellularization, for which we redirect the reader to recent comprehensive reviews [8,9].

## Cellular constituents of advanced human muscle models: beyond primary myoblasts

### Immortalising biopsy-derived skeletal myogenic cells

The ability to culture primary myogenic cells from human skeletal muscle biopsies *ex vivo* is crucial for modelling skeletal muscle function and disease [10,11]. However, the limited availability of patient tissue biopsies and restricted proliferative capacity of the extracted myoblasts make it difficult to use these cells extensively [12]. As a result, several immortalisation strategies have been applied to overcome Hayflick's limit while maintaining the myogenic differentiation potential of isolated primary myoblasts *in vitro*. The most used strategies rely on the dual expression of cell cycle regulators (e.g. CDK4 and Bmi*-*1) and the catalytic subunit of human telomerase [13, 14, 15]. Other strategies include expression of Simian Virus 40 (SV40) large T-antigen [16] and cyclin D1 genes [17] to produce clonal human myogenic cell lines with robust differentiation potential [18,19] that are amenable to genetic manipulation, transplantation, disease modelling and tissue engineering [20, 21, 23]. However, primary cell immortalisation relies on the supply of biopsy-derived myogenic cells which are not always available for disease-specific (e.g. tissue fibrosis or exhaustion of MuSCs in degeneration–regeneration cycles), diagnostic (e.g. fewer muscle biopsies are performed because more diagnoses are being made with genetic testing) and ethical issues (*ad hoc* biopsies for research purposes are not feasible in children with severe muscle disorders). A further concern with primary and immortalised cell lines is their limitation in modelling processes requiring extended time-resolution such as developmental myogenesis of early-onset muscle disorders, given the adult/mature state of the cells [24]. In these cases, pluripotent stem cells (PSCs; including induced PSCs (iPSCs) and embryonic stem cells) provide a particularly useful solution to these hurdles.

### PSC-derived skeletal myogenic cells: lessons from developmental myogenesis

Myogenic differentiation protocols of PSCs take inspiration from biochemical signalling processes that occur during developmental and/or regenerative myogenesis. During embryogenesis, precursor cells for trunk and limb muscles originate from structures of condensed paraxial mesoderm into bilaterally segmented compartments known as somites. Key signalling pathways governing this complex process include those triggered by Sonic hedgehog, Wnt and bone morphogenetic protein produced by the notochord, dorsal neural tube and surface ectoderm and lateral plate mesoderm, respectively [25]. The dermomyotome, an epithelial cell layer at the dorsal end of the somites underneath the ectoderm, is a signalling hotspot for myogenic specification and determination and gives rise to the dermis, skeletal muscle precursor cells, endothelial and vascular smooth muscle cells [26,27]. Crucially, the dorsomedial lip of the dermomyotome is also the site for expression of skeletal myogenic regulatory transcription factors such as myogenic differentiation 1 (MyoD) and myogenic factor 5 (Myf5), that initiate specification of skeletal muscle progenitors [28]. These embryonic myoblasts then migrate under the dermomyotome to form the myotome and then fuse to form embryonic muscle fibres during primary myogenesis. Notably, around this time, Paired box genes 3 and 7 (Pax3/7) positive cells from the dermomyotome migrate into the underlying myotome to sustain muscle growth and establish the future MuSC pool.

Primary myogenesis is followed by foetal or secondary myogenesis (~E14.5-E17.5), characterised by the formation of secondary muscle fibres surrounding the existing primary myofibres and by the onset of innervation. At this stage, MuSCs expressing C-Met, M-Cadherin and Pax7 become identifiable in their characteristic niche between the basal lamina and myofibres (Figure 1b) [26,29]. The MuSCs contribute to the formation of multinucleated fibres by partially fusing with secondary muscle fibres during development. In adult skeletal muscles, MuSCs are normally quiescent and are only activated on injury to first proliferate and then asymmetrically divide into a pool of progenitors that return to quiescence for maintenance and a pool of committed myoblasts that will progressively lose *Pax7* expression while upregulating Myf5 and MyoD [30]. Subsequently, these myoblasts proliferate and fuse with each other and/or other muscle fibres to recover the injured tissue mass in a process that resembles embryonic myogenesis [31].

In the past decade, several methods have been established to differentiate myogenic cells from PSCs (summarised in Table 1). The two main strategies to induce myogenic differentiation of human PSCs are 1) transgene-based, involving the exogenous expression of key myogenic regulators (e.g. Pax3/7 or MyoD) [32,33] sometimes together with epigenetic modulators (e.g. BRG1/BRM-associated factor 60 (BAF60) or Jumonji domain-containing protein D3 (JMJD3)) [34,35] and 2) transgene-free methods which use a cocktail of signalling molecules, growth factors and inhibitors to recapitulate developmental myogenesis [36, 37, 38, 39].

**Table 1:** Overview of key transgene- and small molecule-based skeletal myogenic differentiation studies of human iPSCs/ESCs.

**A. Transgene-mediated**			
**Transgene**	**Cell source**	**Culture method, disease models and remarks**	**References**
MyoD	hiPSC	2D culture, DMD, inducible SMARCD3 gene expression	[35]
	hESC, hiPSC	2D culture, MyoD mRNA transfection and siRNA mediated knockdown of POU5F1	[75]
	hiPSC	Facioscapulohumeral muscular dystrophy, transposon-mediated delivery of tetracycline inducible MyoD	[76]
	hiPSC	2D culture, DMD	[77]
	hiPSC	2D culture, Pompe disease, transposon-mediated delivery of MyoD	[78]
	hiPSC	2D culture, amyotrophic lateral sclerosis, transposon-mediated delivery of MyoD	[79]
	hiPSC	2D culture, epigenetic modulator JMJD3	[34]
	hESC	2D culture, GAG-binding motif for cell penetration peptide	[80]
	hiPSC	2D culture	[81]
	hiPSC	2D culture, exon skipping for DMD	[82]
	hESC, hiPSC	2D culture, limb girdle muscular dystrophy iPSCs, DMD iPSCs. Inducible MyoD expression	[83]
	hiPSC	2D culture, DMD patient hiPSCs for gene correction by TALEN and CRISPR-Cas9	[84]
	hiPSC	EB culture, Carnitine palmitoyltransferase II deficiency patient iPSCs	[85]
	hiPSC	2D culture, DMD patient-derived	[86]
	hESC	Myosphere culture, overexpression of MyoD and BAF60C	[88]
	hiPSC	2D culture, Miyoshi myopathy patient-derived	[89]
	hESC	EB culture, adenoviral delivery	[90]
	hESC	2D culture, Tet-ON system in the lentiviral vector	[91]
	hiPSC	2D culture, limb girdle muscular dystrophy iPSCs, DMD iPSCs, inducible MyoD expression	[33]
	hiPSC	mRNA-mediated	[92]
Pax7	hESC, hiPSC	EB culture	[32]
Pax7	hESC, hiPSC	GSK3-β inhibitor-induced commitment, PAX7-induced differentiation, maturation cocktail	[66]
Myf5	mESC, hESC	EB culture, Lenti-mediated Tet-ON system	[93]

**B. Small molecule-induced**			
**Small molecules and/or culture platform**	**Cell source**	**Culture method and remarks**	**References**
ITS-A, LDN, Wnt activators, BMP inhibitors, CHIR, GSK3 inhibitor, IGF1, HGF, DAPT (notch pathway inhibitor)	hiPSC	2D culture, dual codifferentiation into skeletal muscle cells and motor neurons	[94]
CHIR, LDN, SB431542, HGF, IGF-1	ESC, hiPSC	2D culture	[95]
Wnt activators, TGF-β inhibitors, CHIR, LDN, BMP receptor inhibitors	hiPSC	Sphere-based culture	[96]
FGF2, LY294002, BMP4, CHIR	hESC	2D culture	[97]
GSK3-β inhibitor, ascorbic acid, Alk5 inhibitor, EGF, dexamethasone, insulin	hESC	2D culture	[98]
GSK3-β inhibitor, BMP inhibitor, HGF, IGF, bFGF	hESC, hiPSC	2D culture	[36,99]
GSK3-β inhibitor, BMP, VEGF inhibitor, bFGF	hESC, hiPSC	EB culture	[100]
bFGF, EGF	hESC, hiPSC	Free-floating spherical culture	[101]
GSK3-β inhibitor, CHIR, FGF2	hiPSC	2D culture, FACS sorting	[102,103]
Chitosam-polycaprolactone nanofibres, Wnt3a	hESC	2D culture, C-MET^+^ sorting	[104]
GSK3-β inhibitor, bFGF, forskolin	hiPSC	EB culture	[39]
LiCI, BMP4, activin A	hESC, mESC	2D culture	[105]
–	hESC, hiPSC	EB culture	[106]
TGF-β inhibitor	hESC	GNE−/− EB culture	[107]
OP9 coculture, insulin	hESC	CD73^+^ MSC sorting, NCAM^+^ sorting	[108]
OP9 and C2C12 coculture	hESC	CD73^+^ MSC sorting	[109]

hiPSC, human-induced pluripotent stem cell; hESC, human embryonic stem cell; mESC, mouse embryonic stem cell; DMD, Duchenne muscular dystrophy; EB, embryoid body; 2D, two-dimensional; MSC, mesenchymal stem cell; IGF, insulin growth factor; HGF, hepatocyte growth factor; LDN,LDN193189; CHIR, CHIR99021; ITS, insulin transferrin-selenium; BMP, bone morphogenetic protein; GSK, glycogen synthase kinase; VEGF, vascular endothelial growth factor; FGF, fibroblast growth factor; bFGF, basic FGF; GAG, glycosaminoglycan; JMJD3, jumonji domain-containing protein D3; TGF, transforming growth factor; FACS, fluorescence-activated cell sorting; MSC, mesenchymal stem cell; NCAM, neural cell adhesion molecule; EGF, epidermal growth factor.

Although skeletal muscle models based on monolayer [bidimensional and two-dimensional (2D)] cell cultures are well-established and widely used to study muscle differentiation and disease because they are simple, inexpensive, and user-friendly, their physiological relevance may be limited [40]. Indeed, these 2D models often do not replicate the complexity of the native muscle tissue functional units and niche, where cells of different lineages constantly interact via a range of biochemical and physical factors in different 3D compartments (Figure 1) [41, 42, 43, 44]. To overcome these limitations, muscle biologists have started to exploit the potential of bioengineering to develop 3D human skeletal muscle platforms with a higher degree of complexity and maturation, better resembling native tissues.

## Recapitulating 3D tissue complexity

Strategies to engineer 3D human skeletal muscles can be broadly classified into either 1) self-organised, organoid-like 3D cultures or 2) scaffold-based platforms. Recent notable studies using 3D culture platforms containing human myogenic cells are summarised in Table 2 and discussed in the following sections.

**Table 2:** Summary of significant 3D artificial human skeletal muscle studies since 2018.

Platform	Cell types	Source	Physical cues	Electrical cues	Vascularisation	Functional readout	Disease modelling	References
Organoids on low adhesion plates	Cortical neurons Spinal MNs Skeletal yogenic cells	hiPSCs	-	Optogenetic stimulation	-	Ca^2+^ transients Contraction	-	[46]
	Neuromesodermal progenitors	hPSCs	-	-	-	Ca^2+^ transients Contraction	MG patient antibodies reduce NMJ function	[47]
Cells in hydrogel held between two attachment points	Myoblasts	Human biopsy	Tension along attachment sites	Electrical stimulation	-	Contraction	-	[50]
	Myoblasts	Human biopsy	Tension	EPS	-	Contraction	Reduced α-glucosidase enzyme activity and elevated glycogen content	[51]
	Myoblasts	Human biopsy	Tension	-	-	-	Creatine kinase release	[52]
	Myoblasts	Human biopsy	Tension	EPS	-	Ca^2+^ transients Contraction	Atrophy and lower contractility in senescent muscles	[53]
	Immortalised myoblasts	Human biopsy	Tension	EPS	-	Ca^2+^ imaging Contraction	-	[54]
	Myoblasts	Human biopsy	Tension	Electrical field stimulation	-	Contraction	Regeneration observed after barium chloride injury	[61]
	Myoblasts	Human biopsy	Tension	EPS	-	Contraction	Chemotherapeutic agent reduced contractile force	[74]
	Skeletal myogenic cells MNs Pericytes ECs	hiPSCs	Tension	-	ECs form vessel-like networks in vitro Functional vascularisation upon implantation in mice	Ca^2+^ transients	Engineered muscles from laminopathy patients nuclear abnormalities	[7, 40∗∗]
	Myoblasts Tenocytes	Human biopsy, rat tail	Tension, Spatial bio-printing of tenocytes around post	EPS	-	Ca^2+^ transients	-	[56]
	Myoblasts Fibroblasts MNs	Human biopsy, ESCs	Tension MN spheroids over muscle bundle	Optogenetic stimulation	-	Ca^2+^ transients Contraction	MG antibodies reduced excitability of muscle	[22]
Two compartments of fibrin hydrogel: muscle fibres embedded in endomysium	Immortalised myoblasts Fibroblasts ECs	Human biopsy	Tension Myofibres spatially segregated from fibroblasts + ECs	-	ECs form network of microvessels	-	Fibrosis markers upregulated in Duchenne muscular dystrophic muscles	[55]
Strips of cell-hydrogel bio printed with microchannels	Myoblasts Immortalised neural progenitors	Human biopsy, cell line	Tension (between pillar structures)	Electrical stimulation of peroneal nerve after rodent implantation	Upon implantation	Force measurement of tibialis anterior after implantation	-	[58]
	Myoblasts HUVECs	Human biopsy, cell line	Tension Spatial coaxial bioprinting of myotubes encapsulated by ECs	Electrical stimulation of peroneal nerve after rodent implantation	EC layer Post implantation	Force measurement of tibialis anterior after implantation	-	[59]
Three compartment microfluidic device: myobundle, MN spheroid, EC monolayer	Skeletal myogenic cells Neural stem cells ECs	hiPSCs	Tension between pillars Spatial segregation	Electrical stimulation	EC barrier	Contraction	ALS constructs contracted less and had more MN degradation	[64]
Two compartment BioMEMS device: myoblasts, MNs	Myoblasts MNs	Human biopsy, hiPSCs	Compartments spatially segregated by microtunnels	Electrical stimulation	-	Contraction	-	[62]
Cells in hydrogel bundles anchored by frame structure	Myoblasts	Human biopsy	Tension along attachment sites	Exercise by electrical stimulation	-	Ca^2+^ transients Contraction Acylcarnitine and amino acid levels	-	[68]
	Myoblasts Dermal fibroblasts	Human biopsy	Tension	Exercise by electrical stimulation	-	Contraction	-	[69]
	Myoblasts	Human biopsy	Tension	Exercise-mimetic electrical stimulation	-	Ca^2+^ transients Contraction	Muscle atrophy and proinflammatory cytokine secretion	[70]

hiPSC, human induced pluripotent stem cell; 1°, primary; MG, myasthenia gravis; NMJ, neuromuscular junction; EPS, electrical pulse stimulation; IOPD, infantile-onset Pompe disease; EC, endothelial cell; MN, motor neuron; ESC, embryonic stem cell; ALS, amyotrophic lateral sclerosis; 3D, three-dimensional; HUVEC, human umbilical vein endothelial cells.

### Self-organised 3D skeletal muscle organoids

The principles behind organoid generation could be traced back to Steinberg's differential adhesion hypothesis [45], as per which different cell types tend to segregate themselves based on their adhesive properties. Two recent studies have elegantly shown the generation of human organoids with functional neuromuscular junctions (NMJs) able to stimulate skeletal myofibres via the activation of neuronal circuits [46,47]. Anderson et al. [46] first generated spinal and muscle spheroids before assembling the spheroids together to obtain 3D cortico-motor assembloids, which are complex multicellular models with functional neural circuits. More recently, hiPSC skeletal muscle organoids containing paraxial mesoderm and neuromesodermal progenitors have been induced to foetal hypaxial myogenesis, generating PAX7-positive myogenic and PDGFRa-positive fibroadipogenic progenitor populations which could offer useful inights into human developmental somitogenesis and muscle histogenesis [111]. Although these models provide us with insights into the complexity of muscle tissue and its interface with the neural network (necessary for better modelling of neuromuscular disorders), they do not replicate key architectural features of skeletal muscles such as myofibre alignment, owing to the absence of tension normally provided by tendinous attachments to the bone.

### Scaffold-based platforms to model skeletal muscle tissue architecture

Skeletal muscle is a highly mechanically active tissue undergoing frequent contraction cycles that expose cells, organelles and the surrounding extracellular matrix to physical forces which could in turn impact myogenesis and differentiation. For instance, culturing cells on substrates with physiological rigidity enhances muscle stem cell renewal [43], myogenic differentiation [41] and optimise myotube maturation [48]. Moreover, spatially aligning differentiating myoblasts—either by patterning lines of adhesive protein or by fabricating alternating lines of physiologically stiff and soft hydrogels—further enhances myotube formation and maturation [48,49]. Thus, providing mechanical cues via structural support from a scaffold to cultured myogenic cells is necessary to enhance the physiological relevance of the resulting advanced skeletal muscle models.

Several research groups have successfully created 3D muscle models by embedding differentiating human myoblasts in hydrogels (including fibrin, collagen and Matrigel) anchored between two attachment points [50, 51, 52, 53, 54, 63]. These experimental setups recreate mechanical cues present in the native skeletal muscle niche that are absent in most organoid systems by providing embedded cells with a surrounding matrix that they can attach to, while also presenting an axis of tension in the hydrogel held between the two attachment points that guides myotube alignment. Such tension and alignment of myotubes promote sarcomere maturation and reveal disease-specific phenotypes normally seen with less prevalence in 2D cultures. This was indeed demonstrated by our group for skeletal muscle disorders caused by defective nuclear envelope proteins using patient-specific iPSCs [7,40] with the resulting engineered muscles showing characteristic disease-associated nuclear shape abnormalities secondary to *LMNA* mutations. This finding has been recently validated in an independent study using a miniaturised 3D platform [112]. Other groups have used similar platforms to differentiate primary or iPSC-derived myogenic progenitors to model acute and chronic muscle injuries, disorders and ageing [51, 52, 53, 63] (additional examples are discussed in subsequent sections). Although these culture models recreate the tensional cues from the attachment of muscles to tendons, the majority lacks the multicellular complexity typical of native skeletal muscle tissues as they have been made purely with cells of a single lineage (often using biopsy-derived myoblasts).

### Introducing lineage complexity together with spatial compartmentalisation

Previous work in our group suggests that increasing lineage complexity by including iPSC-derived endothelial cells and pericytes together with myogenic cells (Figure 1c, panel C1) in human 3D skeletal muscle constructs is associated with improved force recovery after injury upon implantation in mice subjected to volumetric muscle injuries [40]. Although cells were not spatially patterned in these constructs, the intrinsic self-organising properties of myotubes and vascular networks resulted in artificial muscles containing vessel-like networks in the matrix surrounding myofibres. Alternatively, cells can be spatially patterned as performed by Bersini et al. [55] (Figure 1c, panel C2), by differentiating myogenic progenitors in fibrin hydrogels and subsequently embedding the muscle fibres in a hydrogel containing endothelial cells and myofibroblasts. The physiological conditions of the skeletal muscle tissue niche reproduced by these constructs made it possible to observe an increased deposition of collagen I and fibronectin in a 3D model of Duchenne muscular dystrophy that could not be seen in 2D. Other notable spatial patterning methods used to create multilineage artificial muscles anchored at two attachment points include the seeding of motor neuron spheroids on top of muscle bundles [22,40] and the bioprinting of tenocytes around post attachment sites with myoblasts in the hydrogel region between posts [56].

Precise spatial patterning of cells and extracellular matrix to create compartmentalised 3D constructs is currently best achieved by bioprinting techniques. Kim et al. [57] used a 3D bioprinting strategy to create aligned strips of myogenic progenitor-laden bioink with hollow microchannels supported by poly(ε-caprolactone) pillars. The organised structure of these constructs enhanced functional recovery, vascularisation and neural integration in the tibialis anterior muscle after implantation into rats. Integrating neural progenitors into the cell bioink layer further improved neuromuscular junction formation and muscle function with reduced signs of fibrosis after implantation [58]. ‘Prevascularised’ muscle constructs printed by a coaxial technique (Figure 1c, panel C3 and 1d), where the strips of the cell-laden hydrogel are spatially segregated with an inner strip of myogenic progenitor bioink encased in a layer of endothelial cell-loaded bioink, further enhanced functional vascularisation and recovery upon implantation [59]. Apart from the benefit to *in vivo* vascularisation, the ability to perfuse muscle constructs through hollow microchannels could have further advantages *in vitro*, such as testing antibody-mediated immune responses (e.g. in myasthenia gravis) or the effects of small-molecule treatments on disease-specific muscle constructs.

### Increasing muscle function with simulated innervation

The complex process of skeletal muscle innervation is simplified *in vitro* by electrical stimulation [60∗∗, 61, 62], chemical treatment [22,40] or optogenetic manipulation [22,46]. Several studies have measured functional parameters of the resulting muscle contraction from such treatments (e.g. Ca^2+^ dynamics and force of contraction). Osaki et al. [64] used this measure to find that artificial muscle microfluidic devices innervated by amyotrophic lateral sclerosis (ALS) iPSC-derived motor neuron spheroids spatially separated from muscle bundles had impaired contraction force compared with control muscles and that the impairment could be partially recovered by treatment with ALS drug candidates.

For diseases such as Duchenne muscular dystrophy where muscles are primarily affected, it is important to generate myotubes that are mature enough to reveal phenotypic readouts for relevant disease modelling. To enhance human skeletal myotube maturation *in vitro* (in terms of gene expression, architecture and contractile ability), cells are usually treated with specific growth factors and small molecules during differentiation [65,66]. Xu et al. [65] showed that exposing myogenic differentiation cultures to endothelial cell growth medium-2 supplements for short time periods enhanced the contractile force generated by myotubes. In another study, Selvaraj et al. [66] used a cocktail of small molecules to enhance myofibril sarcomeric organisation in iPSC-derived myotubes, namely, the transforming growth factor-β (TGF-β) signalling inhibitor SB431542, the γ-secretase and Notch pathway inhibitor DAPT, the glucocorticoid dexamethasone, the MAPK/ERK Kinase (MEK) inhibitor PD0325901 and the adenylyl cyclase activator forskolin. Both studies also demonstrated upregulation of genes (*MYOG* and *MYH3*) and microRNAs (*MIR206* and *MIR113B*) associated with mature muscles.

A way to mimic physiological muscle overuse is to apply long-term electrical field stimulation training. Using this approach, a recent study revealed contractile performance decline in dystrophic iPSC-derived myotubes compared with healthy controls [67]. Electrical stimulation has also been applied to ‘exercise’ artificial 3D muscles with prolonged intermittent electrical stimulation regimes that induce hypertrophy and improve metabolic flux [68]. Takahasi et al. [69] showed that by applying electrical pulse stimulation exercise to myofibre sheets cocultured with dermal fibroblasts, more exercise-related cytokines were released. More recently, advanced muscle models have been used to study the anti-inflammatory effects of muscle exercises using exercise-mimetic electrical stimulation on myobundles made from primary human myoblasts [70]. Applying a similar approach to exercise PSC-derived 3D muscle constructs might further advance the maturation of patient-specific artificial muscles to broaden the spectrum of phenotypic readouts for advanced disease modelling.

## Future perspectives

The aphorism from the statistician George E. P. Box, ‘*all models are wrong, but some are useful*’, concisely summarises the current landscape of cellular modelling of skeletal muscle tissue development, differentiation and disease. Although none of the existing models discussed in this review fully recapitulate all aspects of the physiological skeletal muscle tissue niche, the ability to recreate at least some features has been invaluable to improve our understanding of skeletal muscle growth, disease and regeneration. Excitingly, recent studies are also focussing on closely studying and modelling developmental myogenesis and early (i.e. foetal) muscle disease pathogenesis taking advantage of emerging technologies [37,71,72]. Looking forward, we see the need for better integration of the two main methodologies used to differentiate human iPSCs into functional skeletal myofibres (i.e. transgene- and small molecule-based protocols) alongside the two key strategies to produce artificial skeletal muscle tissues, namely, the organoid systems with scaffold-based 3D culture platforms. Scaffold-based culture platforms (bioprinting in particular [59]) are likely to provide superior structural support and spatial cues more than simpler, self-assembling organoid systems. Regardless of the underlying platform/scaffold, the use of iPSCs makes it possible to obtain a virtually unlimited number of cells from a minimally invasive source to create isogenic (and often isochronic) multilineage tissues for disease modelling, drug development, cell therapy or tissue replacement. Nonetheless, at variance with models based upon non-human cells [110], additional work is required to enhance the maturation of human iPSC-derived platforms: this is particularly relevant to model late-onset diseases, for which the relatively immature myofibres currently generated by the majority of available protocols might not recapitulate phenotypic readouts of adult skeletal muscles with high fidelity. We foresee this problem being rapidly addressed by the field, with promising results already obtained by stimulating cultures *in vitro* chemically [66] or electrically [68]. At the same time, more clinically relevant phenotypic readouts of muscle function need to be consistently measured in these artificial tissues (e.g. creatine kinase release and contraction defects [73]). However, also in this case, suboptimal maturation might pose a challenge. Furthermore, scaling down models without compromising tissue architecture and composition to dimensions amenable to medium-/high-throughput screening platforms will become increasingly important in the next decade, and progress is also being made on that front [74, 112]. Close multidisciplinary collaborations between muscle biologists, tissue engineers and clinicians are likely to provide solutions to address all the aforementioned challenges in the near future.

## Conflict of interest statement

FST provides consulting services to Aleph Farms via UCL Consultants. The other authors do not declare conflict of interest.

## References

[1] Frontera W.R., Ochala J. (2015). Skeletal muscle: a brief review of structure and function. Calcif Tissue Int.

[2] Csapo R., Gumpenberger M., Wessner B. (2020). Skeletal muscle extracellular matrix – what do we know about its composition, regulation, and physiological roles? A narrative review. Front Physiol.

[3] Christov C., Chrétien F., Abou-Khalil R., Bassez G., Vallet G., Authier F.-J., Bassaglia Y., Shinin V., Tajbakhsh S., Chazaud B. (2007). Muscle satellite cells and endothelial cells: close neighbors and privileged partners. Mol Biol Cell.

[4] Ratnayake D., Nguyen P.D., Rossello F.J., Wimmer V.C., Tan J.L., Galvis L.A., Julier Z., Wood A.J., Boudier T., Isiaku A.I. (2021). Macrophages provide a transient muscle stem cell niche via NAMPT secretion. Nature.

[5] Mercuri E., Bönnemann C.G., Muntoni F. (2019). Muscular dystrophies. Lancet.

[6] van Putten M., Putker K., Overzier M., Adamzek W.A., Pasteuning-Vuhman S., Plomp J.J., Aartsma-Rus A. (2019). Natural disease history of the D2-mdx mouse model for Duchenne muscular dystrophy. Faseb J.

[7] Steele-Stallard H.B., Pinton L., Sarcar S., Ozdemir T., Maffioletti S.M., Zammit P.S., Tedesco F.S. (2018). Modeling skeletal muscle laminopathies using human induced pluripotent stem cells carrying pathogenic LMNA mutations. Front Physiol.

[8] Urciuolo A., De Coppi P. (2018). Decellularized tissue for muscle regeneration. Int J Mol Sci.

[9] McCrary M.W., Bousalis D., Mobini S., Song Y.H., Schmidt C.E. (2020). Decellularized tissues as platforms for in vitro modeling of healthy and diseased tissues. Acta Biomater.

[10] Pietrangelo T., Puglielli C., Mancinelli R., Beccafico S., Fanò G., Fulle S. (2009). Molecular basis of the myogenic profile of aged human skeletal muscle satellite cells during differentiation. Exp Gerontol.

[11] Abdelmoez A.M., Sardón Puig L., Smith J.A.B., Gabriel B.M., Savikj M., Dollet L., Chibalin A.V., Krook A., Zierath J.R., Pillon N.J. (2019). Comparative profiling of skeletal muscle models reveals heterogeneity of transcriptome and metabolism. Am J Physiol Cell Physiol.

[12] Massenet J., Gitiaux C., Magnan M., Cuvellier S., Hubas A., Nusbaum P., Dilworth F.J., Desguerre I., Chazaud B. (2020). Derivation and characterization of immortalized human muscle satellite cell clones from muscular dystrophy patients and healthy individuals. Cells.

[13] Zhu C.-H., Mouly V., Cooper R.N., Mamchaoui K., Bigot A., Shay J.W., Di Santo J.P., Butler-Browne G.S., Wright W.E. (2007). Cellular senescence in human myoblasts is overcome by human telomerase reverse transcriptase and cyclin-dependent kinase 4: consequences in aging muscle and therapeutic strategies for muscular dystrophies. Aging Cell.

[14] Cudré-Mauroux C., Occhiodoro T., König S., Salmon P., Bernheim L., Trono D. (2003). Lentivector-mediated transfer of Bmi-1 and telomerase in muscle satellite cells yields a duchenne myoblast cell line with long-term genotypic and phenotypic stability. Hum Gene Ther.

[15] Halvorsen T.L., Leibowitz G., Levine F. (1999). Telomerase activity is sufficient to allow transformed cells to escape from crisis. Mol Cell Biol.

[16] Simon L.V., Beauchamp J.R., O'Hare M., Olsen I. (1996). Establishment of long-term myogenic cultures from patients with duchenne muscular dystrophy by retroviral transduction of a temperature-sensitive SV40 large T antigen. Exp Cell Res.

[17] Shiomi K., Kiyono T., Okamura K., Uezumi M., Goto Y., Yasumoto S., Shimizu S., Hashimoto N. (2011). CDK4 and cyclin D1 allow human myogenic cells to recapture growth property without compromising differentiation potential. Gene Ther.

[18] Thorley M., Duguez S., Mazza E.M.C., Valsoni S., Bigot A., Mamchaoui K., Harmon B., Voit T., Mouly V., Duddy W. (2016). Skeletal muscle characteristics are preserved in hTERT/cdk4 human myogenic cell lines. Skeletal Muscle.

[19] Pantic B., Borgia D., Giunco S., Malena A., Kiyono T., Salvatori S., De Rossi A., Giardina E., Sangiuolo F., Pegoraro E. (2016). Reliable and versatile immortal muscle cell models from healthy and myotonic dystrophy type 1 primary human myoblasts. Exp Cell Res.

[20] Benedetti S., Uno N., Hoshiya H., Ragazzi M., Ferrari G., Kazuki Y., Moyle L.A., Tonlorenzi R., Lombardo A., Chaouch S. (2018). Reversible immortalisation enables genetic correction of human muscle progenitors and engineering of next-generation human artificial chromosomes for Duchenne muscular dystrophy. EMBO Mol Med.

[21] Prüller J., Mannhardt I., Eschenhagen T., Zammit P.S., Figeac N. (2018). Satellite cells delivered in their niche efficiently generate functional myotubes in three-dimensional cell culture. PloS One.

[bib22] Afshar Bakooshli M., Lippmann E.S., Mulcahy B., Iyer N., Nguyen C.T., Tung K., Stewart B.A., Van Den Dorpel H., Fuehrmann T., Shoichet M. (2019). A 3D culture model of innervated human skeletal muscle enables studies of the adult neuromuscular junction. eLife.

[23] Mamchaoui K., Trollet C., Bigot A., Negroni E., Chaouch S., Wolff A., Kandalla P.K., Marie S., Di Santo J., St Guily J.L. (2011). Immortalized pathological human myoblasts: towards a universal tool for the study of neuromuscular disorders. Skeletal Muscle.

[24] Kimmel J.C., Yi N., Roy M., Hendrickson D.G., Kelley D.R. (2021). Differentiation reveals latent features of aging and an energy barrier in murine myogenesis. Cell Rep.

[25] Ordahl C.P., Le Douarin N.M. (1992). Two myogenic lineages within the developing somite. Development.

[26] Kassar-Duchossoy L., Giacone E., Gayraud-Morel B., Jory A., Gomès D., Tajbakhsh S. (2005). Pax3/Pax7 mark a novel population of primitive myogenic cells during development. Gene Dev.

[27] Relaix F., Rocancourt D., Mansouri A., Buckingham M. (2005). A Pax3/Pax7-dependent population of skeletal muscle progenitor cells. Nature.

[28] Rudnicki M.A., Schnegelsberg P.N.J., Stead R.H., Braun T., Arnold H.-H., Jaenisch R. (1993). MyoD or Myf-5 is required for the formation of skeletal muscle. Cell.

[29] Mauro A. (1961). Satellite cell of skeletal muscle fibers. J Biophys Biochem Cytol.

[30] Cooper R.N., Tajbakhsh S., Mouly V., Cossu G., Buckingham M., Butler-Browne G.S. (1999). In vivo satellite cell activation via Myf5 and MyoD in regenerating mouse skeletal muscle. J Cell Sci.

[31] Schiaffino S., Rossi A.C., Smerdu V., Leinwand L.A., Reggiani C. (2015). Developmental myosins: expression patterns and functional significance. Skeletal Muscle.

[32] Darabi R., Arpke Robert W., Irion S., Dimos John T., Grskovic M., Kyba M., Perlingeiro R. (2012). Human ES- and iPS-derived myogenic progenitors restore DYSTROPHIN and improve contractility upon transplantation in dystrophic mice. Cell Stem Cell.

[33] Tedesco F.S., Gerli M.F.M., Perani L., Benedetti S., Ungaro F., Cassano M., Antonini S., Tagliafico E., Artusi V., Longa E. (2012). Transplantation of genetically corrected human iPSC-derived progenitors in mice with limb-girdle muscular dystrophy. Sci Transl Med.

[34] Akiyama T., Wakabayashi S., Soma A., Sato S., Nakatake Y., Oda M., Murakami M., Sakota M., Chikazawa-Nohtomi N., Ko S.B.H. (2016). Transient ectopic expression of the histone demethylase JMJD3 accelerates the differentiation of human pluripotent stem cells. Development.

[35] Caputo L., Granados A., Lenzi J., Rosa A., Ait-Si-Ali S., Puri P.L., Albini S. (2020). Acute conversion of patient-derived Duchenne muscular dystrophy iPSC into myotubes reveals constitutive and inducible over-activation of TGFβ-dependent pro-fibrotic signaling. Skeletal Muscle.

[36] Chal J., Al Tanoury Z., Hestin M., Gobert B., Aivio S., Hick A., Cherrier T., Nesmith A.P., Parker K.K., Pourquié O. (2016). Generation of human muscle fibers and satellite-like cells from human pluripotent stem cells in vitro. Nat Protoc.

[37] Xi H., Langerman J., Sabri S., Chien P., Young C.S., Younesi S., Hicks M., Gonzalez K., Fujiwara W., Marzi J. (2020). A human skeletal muscle atlas identifies the trajectories of stem and progenitor cells across development and from human pluripotent stem cells. Cell Stem Cell.

[38] Nalbandian M., Zhao M., Sasaki-Honda M., Jonouchi T., Lucena-Cacace A., Mizusawa T., Yasuda M., Yoshida Y., Hotta A., Sakurai H. (2021). Characterization of hiPSC-derived muscle progenitors reveals distinctive markers for myogenic cell purification toward cell therapy. Stem Cell Rep.

[39] Xu C., Tabebordbar M., Iovino S., Ciarlo C., Liu J., Castiglioni A., Price E., Liu M., Barton Elisabeth R., Kahn C.R. (2013). A zebrafish embryo culture system defines factors that promote vertebrate myogenesis across species. Cell.

[bib40] Maffioletti S.M., Sarcar S., Henderson A.B.H., Mannhardt I., Pinton L., Moyle L.A., Steele-Stallard H., Cappellari O., Wells K.E., Ferrari G. (2018). Three-dimensional human iPSC-derived artificial skeletal muscles model muscular dystrophies and enable multilineage tissue engineering. Cell Rep.

[41] Engler A.J., Sen S., Sweeney H.L., Discher D.E. (2006). Matrix elasticity directs stem cell lineage specification. Cell.

[42] Engler A.J., Griffin M.A., Sen S., BöNnemann C.G., Sweeney H.L., Discher D.E. (2004). Myotubes differentiate optimally on substrates with tissue-like stiffness: pathological implications for soft or stiff microenvironments. J Cell Biol.

[43] Gilbert P.M., Havenstrite K.L., Magnusson K.E.G., Sacco A., Leonardi N.A., Kraft P., Nguyen N.K., Thrun S., Lutolf M.P., Blau H.M. (2010). Substrate elasticity regulates skeletal muscle stem cell self-renewal in culture. Science.

[44] Soares C.P., Midlej V., MEWd Oliveira, Benchimol M., Costa M.L., Mermelstein C. (2012). 2D and 3D-organized cardiac cells shows differences in cellular morphology, adhesion junctions, presence of myofibrils and protein expression. PloS One.

[45] Steinberg M.S., Locke M. (1964). Cellular membranes in development.

[bib46] Andersen J., Revah O., Miura Y., Thom N., Amin N.D., Kelley K.W., Singh M., Chen X., Thete M.V., Walczak E.M. (2020). Generation of functional human 3D cortico-motor assembloids. Cell.

[bib47] Faustino Martins J.-M., Fischer C., Urzi A., Vidal R., Kunz S., Ruffault P.-L., Kabuss L., Hube I., Gazzerro E., Birchmeier C. (2020). Self-organizing 3D human trunk neuromuscular organoids. Cell Stem Cell.

[48] Jiwlawat N., Lynch E.M., Napiwocki B.N., Stempien A., Ashton R.S., Kamp T.J., Crone W.C., Suzuki M. (2019). Micropatterned substrates with physiological stiffness promote cell maturation and Pompe disease phenotype in human induced pluripotent stem cell-derived skeletal myocytes. Biotechnol Bioeng.

[49] Young J., Margaron Y., Fernandes M., Duchemin-Pelletier E., Michaud J., Flaender M., Lorintiu O., Degot S., Poydenot P. (2018). MyoScreen, a high-throughput phenotypic screening platform enabling muscle drug discovery. SLAS Discov: Adv Sci Drug Discov.

[50] Capel A.J., Rimington R.P., Fleming J.W., Player D.J., Baker L.A., Turner M.C., Jones J.M., Martin N.R.W., Ferguson R.A., Mudera V.C. (2019). Scalable 3D printed molds for human tissue engineered skeletal muscle. Front Bioeng Biotechnol.

[51] Wang J., Zhou C.J., Khodabukus A., Tran S., Han S.-O., Carlson A.L., Madden L., Kishnani P.S., Koeberl D.D., Bursac N. (2021). Three-dimensional tissue-engineered human skeletal muscle model of Pompe disease. Commun Biol.

[52] Gholobova D., Gerard M., Decroix L., Desender L., Callewaert N., Annaert P., Thorrez L. (2018). Human tissue-engineered skeletal muscle: a novel 3D in vitro model for drug disposition and toxicity after intramuscular injection. Sci Rep.

[53] Rajabian N., Shahini A., Asmani M., Vydiam K., Choudhury D., Nguyen T., Ikhapoh I., Zhao R., Lei P., Andreadis S.T. (2020). Bioengineered skeletal muscle as a model of muscle aging and regeneration. Tissue Eng.

[54] Shima A., Morimoto Y., Sweeney H.L., Takeuchi S. (2018). Three-dimensional contractile muscle tissue consisting of human skeletal myocyte cell line. Exp Cell Res.

[bib55] Bersini S., Gilardi M., Ugolini G.S., Sansoni V., Talò G., Perego S., Zanotti S., Ostano P., Mora M., Soncini M. (2018). Engineering an environment for the study of fibrosis: a 3D human muscle model with Endothelium specificity and endomysium. Cell Rep.

[56] Laternser S., Keller H., Leupin O., Rausch M., Graf-Hausner U., Rimann M. (2018). A novel microplate 3D bioprinting platform for the engineering of muscle and tendon tissues. SLAS Technol: Transl Life Sci Innovat.

[57] Kim J.H., Seol Y.-J., Ko I.K., Kang H.-W., Lee Y.K., Yoo J.J., Atala A., Lee S.J. (2018). 3D bioprinted human skeletal muscle constructs for muscle function restoration. Sci Rep.

[bib58] Kim J.H., Kim I., Seol Y.-J., Ko I.K., Yoo J.J., Atala A., Lee S.J. (2020). Neural cell integration into 3D bioprinted skeletal muscle constructs accelerates restoration of muscle function. Nat Commun.

[bib59] Choi Y.-J., Jun Y.-J., Kim D.Y., Yi H.-G., Chae S.-H., Kang J., Lee J., Gao G., Kong J.-S., Jang J. (2019). A 3D cell printed muscle construct with tissue-derived bioink for the treatment of volumetric muscle loss. Biomaterials.

[bib60] Rao L., Qian Y., Khodabukus A., Ribar T., Bursac N. (2018). Engineering human pluripotent stem cells into a functional skeletal muscle tissue. Nat Commun.

[61] Fleming J.W., Capel A.J., Rimington R.P., Wheeler P., Leonard A.N., Bishop N.C., Davies O.G., Lewis M.P. (2020). Bioengineered human skeletal muscle capable of functional regeneration. BMC Biol.

[62] Santhanam N., Kumanchik L., Guo X., Sommerhage F., Cai Y., Jackson M., Martin C., Saad G., McAleer C.W., Wang Y. (2018). Stem cell derived phenotypic human neuromuscular junction model for dose response evaluation of therapeutics. Biomaterials.

[63] Ebrahimi M., Lad H., Fusto A., Tiper Y., Datye A., Nguyen C.T., Jacques E., Moyle L.A., Nguyen T., Musgrave B. (2021). De novo revertant fiber formation and therapy testing in a 3D culture model of Duchenne muscular dystrophy skeletal muscle. Acta Biomater.

[bib64] Osaki T., Uzel S.G.M., Kamm R.D. (2018). Microphysiological 3D model of amyotrophic lateral sclerosis (ALS) from human iPS-derived muscle cells and optogenetic motor neurons. Sci Adv.

[65] Xu B., Zhang M., Perlingeiro R.C.R., Shen W. (2019). Skeletal muscle constructs engineered from human embryonic stem cell derived myogenic progenitors exhibit enhanced contractile forces when differentiated in a medium containing EGM-2 supplements. Adv Biosys.

[66] Selvaraj S., Mondragon-Gonzalez R., Xu B., Magli A., Kim H., Lainé J., Kiley J., McKee H., Rinaldi F., Aho J. (2019). Screening identifies small molecules that enhance the maturation of human pluripotent stem cell-derived myotubes. eLife.

[67] Uchimura T., Asano T., Nakata T., Hotta A., Sakurai H. (2021). A muscle fatigue-like contractile decline was recapitulated using skeletal myotubes from Duchenne muscular dystrophy patient-derived iPSCs. Cell Rep Med.

[68] Khodabukus A., Madden L., Prabhu N.K., Koves T.R., Jackman C.P., Muoio D.M., Bursac N. (2019). Electrical stimulation increases hypertrophy and metabolic flux in tissue-engineered human skeletal muscle. Biomaterials.

[69] Takahashi H., Shimizu T., Okano T. (2018). Engineered human contractile myofiber sheets as a platform for studies of skeletal muscle physiology. Sci Rep.

[70] Chen Z., Li B., Zhan R.-Z., Rao L., Bursac N. (2021). Exercise mimetics and JAK inhibition attenuate IFN-γ–induced wasting in engineered human skeletal muscle. Sci Adv.

[71] Mournetas V., Massouridès E., Dupont J.-B., Kornobis E., Polvèche H., Jarrige M., Dorval A.R.L., Gosselin M.R.F., Manousopoulou A., Garbis S.D. (2021). Myogenesis modelled by human pluripotent stem cells: a multi-omic study of Duchenne myopathy early onset. J Cachexia, Sarcopenia Muscle.

[72] Budjan C., Liu S., Ranga A., Gayen S., Pourquie O., Hormoz S. (2021). Paraxial mesoderm organoids model development of human somites. bioRxiv.

[73] Tanoury Z.A., Zimmermann J.F., Rao J., Sieiro D., McNamara H., Cherrier T., Hick A., Bousson F., Fugier C., Marchiano F. (2020). Prednisolone rescues Duchenne Muscular Dystrophy phenotypes in human pluripotent stem cells-derived skeletal muscle <em>in vitro</em&gt. bioRxiv.

[74] Afshar M.E., Abraha H.Y., Bakooshli M.A., Davoudi S., Thavandiran N., Tung K., Ahn H., Ginsberg H.J., Zandstra P.W., Gilbert P.M. (2020). A 96-well culture platform enables longitudinal analyses of engineered human skeletal muscle microtissue strength. Sci Rep.

[75] Akiyama T., Sato S., Chikazawa-Nohtomi N., Soma A., Kimura H., Wakabayashi S., Ko S.B.H., Ko M.S.H. (2018). Efficient differentiation of human pluripotent stem cells into skeletal muscle cells by combining RNA-based MYOD1-expression and POU5F1-silencing. Sci Rep.

[76] Sasaki-Honda M., Jonouchi T., Arai M., Hotta A., Mitsuhashi S., Nishino I., Matsuda R., Sakurai H. (2018). A patient-derived iPSC model revealed oxidative stress increases facioscapulohumeral muscular dystrophy-causative DUX4. Hum Mol Genet.

[77] Zhao M., Shoji E., Sakurai H., Yokota T., Maruyama R. (2018). Exon skipping and inclusion therapies: methods and protocols.

[78] Yoshida T., Awaya T., Jonouchi T., Kimura R., Kimura S., Era T., Heike T., Sakurai H. (2017). A skeletal muscle model of infantile-onset Pompe disease with patient-specific iPS cells. Sci Rep.

[79] Lenzi J., Pagani F., De Santis R., Limatola C., Bozzoni I., Di Angelantonio S., Rosa A. (2016). Differentiation of control and ALS mutant human iPSCs into functional skeletal muscle cells, a tool for the study of neuromuscolar diseases. Stem Cell Res.

[80] Dixon J.E., Osman G., Morris G.E., Markides H., Rotherham M., Bayoussef Z. (2016). El Haj AJ, Denning C, Shakesheff KM: highly efficient delivery of functional cargoes by the synergistic effect of GAG binding motifs and cell-penetrating peptides. Proc Natl Acad Sci U S A.

[81] Shoji E., Woltjen K., Sakurai H. (2016). Directed myogenic differentiation of human induced pluripotent stem cells. Methods Mol Biol.

[82] Shoji E., Sakurai H., Nishino T., Nakahata T., Heike T., Awaya T., Fujii N., Manabe Y., Matsuo M., Sehara-Fujisawa A. (2015). Early pathogenesis of Duchenne muscular dystrophy modelled in patient-derived human induced pluripotent stem cells. Sci Rep.

[83] Maffioletti S.M., Gerli M.F., Ragazzi M., Dastidar S., Benedetti S., Loperfido M., VandenDriessche T., Chuah M.K., Tedesco F.S. (2015). Efficient derivation and inducible differentiation of expandable skeletal myogenic cells from human ES and patient-specific iPS cells. Nat Protoc.

[84] Li H.L., Fujimoto N., Sasakawa N., Shirai S., Ohkame T., Sakuma T., Tanaka M., Amano N., Watanabe A., Sakurai H. (2015). Precise correction of the dystrophin gene in Duchenne muscular dystrophy patient induced pluripotent stem cells by TALEN and CRISPR-Cas9. Stem Cell Rep.

[85] Yasuno T., Osafune K., Sakurai H., Asaka I., Tanaka A., Yamaguchi S., Yamada K., Hitomi H., Arai S., Kurose Y. (2014). Functional analysis of iPSC-derived myocytes from a patient with carnitine palmitoyltransferase II deficiency. Biochem Biophys Res Commun.

[86] Abujarour R., Bennett M., Valamehr B., Lee T.T., Robinson M., Robbins D., Le T., Lai K., Flynn P. (2014). Myogenic differentiation of muscular dystrophy-specific induced pluripotent stem cells for use in drug discovery. Stem Cells Transl Med.

[88] Albini S., Coutinho P., Malecova B., Giordani L., Savchenko A., Forcales S.V., Puri P.L. (2013). Epigenetic reprogramming of human embryonic stem cells into skeletal muscle cells and generation of contractile myospheres. Cell Rep.

[89] Tanaka A., Woltjen K., Miyake K., Hotta A., Ikeya M., Yamamoto T., Nishino T., Shoji E., Sehara-Fujisawa A., Manabe Y. (2013). Efficient and reproducible myogenic differentiation from human iPS cells: prospects for modeling Miyoshi Myopathy in vitro. PloS One.

[90] Goudenege S., Lebel C., Huot N.B., Dufour C., Fujii I., Gekas J., Rousseau J., Tremblay J.P. (2012). Myoblasts derived from normal hESCs and dystrophic hiPSCs efficiently fuse with existing muscle fibers following transplantation. Mol Ther.

[91] Rao L., Tang W., Wei Y., Bao L., Chen J., Chen H., He L., Lu P., Ren J., Wu L. (2012). Highly efficient derivation of skeletal myotubes from human embryonic stem cells. Stem Cell Rev Rep.

[92] Warren L., Manos P.D., Ahfeldt T., Loh Y.H., Li H., Lau F., Ebina W., Mandal P.K., Smith Z.D., Meissner A. (2010). Highly efficient reprogramming to pluripotency and directed differentiation of human cells with synthetic modified mRNA. Cell Stem Cell.

[93] Iacovino M., Bosnakovski D., Fey H., Rux D., Bajwa G., Mahen E., Mitanoska A., Xu Z., Kyba M. (2011). Inducible cassette exchange: a rapid and efficient system enabling conditional gene expression in embryonic stem and primary cells. Stem Cell.

[94] Mazaleyrat K., Badja C., Broucqsault N., Chevalier R., Laberthonnière C., Dion C., Baldasseroni L., El-Yazidi C., Thomas M., Bachelier R. (2020). Multilineage differentiation for formation of innervated skeletal muscle fibers from healthy and diseased human pluripotent stem cells. Cells.

[95] Xi H., Fujiwara W., Gonzalez K., Jan M., Liebscher S., Van Handel B., Schenke-Layland K., Pyle A.D. (2017). Vivo human somitogenesis guides somite development from hPSCs. Cell Rep.

[96] Sakai-Takemura F., Narita A., Masuda S., Wakamatsu T., Watanabe N., Nishiyama T., Ki Nogami, Blanc M., Si Takeda, Miyagoe-Suzuki Y. (2018). Premyogenic progenitors derived from human pluripotent stem cells expand in floating culture and differentiate into transplantable myogenic progenitors. Sci Rep.

[97] Swartz E.W., Baek J., Pribadi M., Wojta K.J., Almeida S., Karydas A., Gao F.-B., Miller B.L., Coppola G. (2016). A novel protocol for directed differentiation of C9orf72-associated human induced pluripotent stem cells into contractile skeletal myotubes. STEM CELLS Transl Med.

[98] Caron L., Kher D., Lee K.L., McKernan R., Dumevska B., Hidalgo A., Li J., Yang H., Main H., Ferri G. (2016). A human pluripotent stem cell model of facioscapulohumeral muscular dystrophy-affected skeletal muscles. Stem Cells Transl Med.

[99] Chal J., Oginuma M., Al Tanoury Z., Gobert B., Sumara O., Hick A., Bousson F., Zidouni Y., Mursch C., Moncuquet P. (2015). Differentiation of pluripotent stem cells to muscle fiber to model Duchenne muscular dystrophy. Nat Biotechnol.

[100] Shelton M., Metz J., Liu J., Carpenedo R.L., Demers S.P., Stanford W.L., Skerjanc I.S. (2014). Derivation and expansion of PAX7-positive muscle progenitors from human and mouse embryonic stem cells. Stem Cell Rep.

[101] Hosoyama T., McGivern J.V., Van Dyke J.M., Ebert A.D., Suzuki M. (2014). Derivation of myogenic progenitors directly from human pluripotent stem cells using a sphere-based culture. Stem Cells Transl Med.

[102] Borchin B., Chen J., Barberi T. (2013). Derivation and FACS-mediated purification of PAX3+/PAX7+ skeletal muscle precursors from human pluripotent stem cells. Stem Cell Rep.

[103] van der Wal E., Herrero-Hernandez P., Wan R., Broeders M., in 't Groen S.L.M., van Gestel T.J.M., van Ijcken W.F.J., Cheung T.H., van der Ploeg A.T., Schaaf G.J. (2018). Large-scale expansion of human iPSC-derived skeletal muscle cells for disease modeling and cell-based therapeutic strategies. Stem Cell Rep.

[104] Leung M., Cooper A., Jana S., Tsao C.T., Petrie T.A., Zhang M. (2013). Nanofiber-based in vitro system for high myogenic differentiation of human embryonic stem cells. Biomacromolecules.

[105] Sakurai H., Sakaguchi Y., Shoji E., Nishino T., Maki I., Sakai H., Hanaoka K., Kakizuka A., Sehara-Fujisawa A. (2012). In vitro modeling of paraxial mesodermal progenitors derived from induced pluripotent stem cells. PloS One.

[106] Awaya T., Kato T., Mizuno Y., Chang H., Niwa A., Umeda K., Nakahata T., Heike T. (2012). Selective development of myogenic mesenchymal cells from human embryonic and induced pluripotent stem cells. PloS One.

[107] Teng H.F., Kuo Y.L., Loo M.R., Li C.L., Chu T.W., Suo H., Liu H.S., Lin K.H., Chen S.L. (2010). Valproic acid enhances Oct4 promoter activity in myogenic cells. J Cell Biochem.

[108] Barberi T., Bradbury M., Dincer Z., Panagiotakos G., Socci N.D., Studer L. (2007). Derivation of engraftable skeletal myoblasts from human embryonic stem cells. Nat Med.

[109] Barberi T., Willis L.M., Socci N.D., Studer L. (2005). Derivation of multipotent mesenchymal precursors from human embryonic stem cells. PLoS Med.

[110] Santoso J.W., Li X., Gupta D., Suh G.C., Hendricks E., Lin S., Perry S., Ichida J.K., Dickman D., McCain M.L. (2021). Engineering skeletal muscle tissues with advanced maturity improves synapse formation with human induced pluripotent stem cell-derived motor neurons. APL Bioeng.

[111] Mavrommatis L., Jeong H-W., Gomez-Giro G., Stehling M., Kienitz M-C., Psathaki O.E., Bixel M.G., Morosan-Puopolo G., Gerovska D., Araúzo-Bravo M.J., Schwamborn J.C., Schöler H.R., Adams R.H., Vorgerd M., Brand-Saberi B., Zaehres H. (2021). Human skeletal muscle organoids model fetal myogenesis and sustain uncommitted PAX7 myogenic progenitors. bioRxiv.

[112] Rose N., Sonam S., Nguyen T., Grenci G., Bigot A., Muchir A., Ladoux B., Le Grand F., Trichet L. (2021). Bioengineering a miniaturized in vitro 3D myotube contraction monitoring chip for modelization of muscular dystrophies. bioRxiv.

